# Supplementary material for: COZOID: contact zone identifier for visual analysis of protein-protein interactions
Source: BMC Bioinformatics. 2018 Apr 6;19:125. doi: 10.1186/s12859-018-2113-6 (PMC5889581; doi:10.1186/s12859-018-2113-6)
Supplement: Supplementary file 2 — Software build. Executable binary file of the software tool. (ZIP 118,784 kb) [file 12859_2018_2113_MOESM2_ESM.zip › cozoid/platform/docs/testng-6.5.1-javadoc.zip!/org/testng/Reporter.html]

Reporter (TestNG)


---


|  |  |  |
| --- | --- | --- |
| **Overview** \| **Package** \| **Class** \| **Tree** \| **Deprecated** \| **Index** \| **Help** | |  |
| **PREV CLASS**   **NEXT CLASS** | **FRAMES**    **NO FRAMES**     **All Classes** |  |
| SUMMARY: NESTED \| FIELD \| CONSTR \| METHOD | DETAIL: FIELD \| CONSTR \| METHOD |  |


---


## org.testng Class Reporter

```
java.lang.Object
  org.testng.Reporter
```

---

``` public class Reporter extends java.lang.Object ```

This class is used for test methods to log messages that will be
included in the HTML reports generated by TestNG.
  
  
**Implementation details.**
  
  
The reporter keeps a combined output of strings (in m\_output) and also
a record of which method output which line. In order to do this, callers
specify what the current method is with setCurrentMethod() and the
Reporter maintaing a mapping of each method with a list of integers.
These integers are indices in the combined output (avoids duplicating
the output).
Created on Nov 2, 2005

**Author:**
:   cbeust

---

**Constructor Summary**

`Reporter()`


| **Method Summary** | |
| --- | --- |
| `static void` | `clear()`             Erase the content of all the output generated so far. |
| `static ITestResult` | `getCurrentTestResult()` |
| `static java.util.List<java.lang.String>` | `getOutput()` |
| `static java.util.List<java.lang.String>` | `getOutput(ITestResult tr)` |
| `static void` | `log(java.lang.String s)`             Log the passed string to the HTML reports |
| `static void` | `log(java.lang.String s, boolean logToStandardOut)`             Log the passed string to the HTML reports. |
| `static void` | `log(java.lang.String s, int level)`             Log the passed string to the HTML reports if the current verbosity is equal or greater than the one passed in parameter |
| `static void` | `log(java.lang.String s, int level, boolean logToStandardOut)`             Log the passed string to the HTML reports if the current verbosity is equal or greater than the one passed in parameter. |
| `static void` | `setCurrentTestResult(ITestResult m)` |

**Methods inherited from class java.lang.Object**

`clone, equals, finalize, getClass, hashCode, notify, notifyAll, toString, wait, wait, wait`

**Constructor Detail**

### Reporter

```
public Reporter()
```


**Method Detail**

### setCurrentTestResult

```
public static void setCurrentTestResult(ITestResult m)
```

---


### getOutput

```
public static java.util.List<java.lang.String> getOutput()
```

---


### clear

```
public static void clear()
```

:   Erase the content of all the output generated so far.

---


### log

```
public static void log(java.lang.String s)
```

:   Log the passed string to the HTML reports

    :   **Parameters:**: `s` - The message to log

---


### log

```
public static void log(java.lang.String s,
                       int level,
                       boolean logToStandardOut)
```

:   Log the passed string to the HTML reports if the current verbosity
    is equal or greater than the one passed in parameter. If logToStandardOut
    is true, the string will also be printed on standard out.

    :   **Parameters:**: `s` - The message to log: `level` - The verbosity of this message: `logToStandardOut` - Whether to print this string on standard out too

---


### log

```
public static void log(java.lang.String s,
                       boolean logToStandardOut)
```

:   Log the passed string to the HTML reports. If logToStandardOut
    is true, the string will also be printed on standard out.

    :   **Parameters:**: `s` - The message to log: `logToStandardOut` - Whether to print this string on standard out too

---


### log

```
public static void log(java.lang.String s,
                       int level)
```

:   Log the passed string to the HTML reports if the current verbosity
    is equal or greater than the one passed in parameter

    :   **Parameters:**: `s` - The message to log: `level` - The verbosity of this message

---


### getCurrentTestResult

```
public static ITestResult getCurrentTestResult()
```

:   **Returns:**: the current test result.

---


### getOutput

```
public static java.util.List<java.lang.String> getOutput(ITestResult tr)
```


---


|  |  |  |
| --- | --- | --- |
| **Overview** \| **Package** \| **Class** \| **Tree** \| **Deprecated** \| **Index** \| **Help** | |  |
| **PREV CLASS**   **NEXT CLASS** | **FRAMES**    **NO FRAMES**     **All Classes** |  |
| SUMMARY: NESTED \| FIELD \| CONSTR \| METHOD | DETAIL: FIELD \| CONSTR \| METHOD |  |


---
